# Effect of Gliadin on Permeability of Intestinal Biopsy Explants from Celiac Disease Patients and Patients with Non-Celiac Gluten Sensitivity

**DOI:** 10.3390/nu7031565

**Published:** 2015-02-27

**Authors:** Justin Hollon, Elaine Leonard Puppa, Bruce Greenwald, Eric Goldberg, Anthony Guerrerio, Alessio Fasano

**Affiliations:** 1 Department of Pediatric Gastroenterology, Naval Medical Center Portsmouth, 620 John Paul Jones Circle, Portsmouth, VA 23708, USA; 2 University of Maryland School of Medicine, Baltimore, MD 21201, USA; E-Mail: eleonard@peds.umaryland.edu; 3 Division of Gastroenterology and Hepatology, University of Maryland School of Medicine, Baltimore, MD 21201, USA; E-Mails: bgreenwa@medicine.umaryland.edu (B.G.); egoldber@medicine.umaryland.edu (E.G.); 4 Division of Pediatric Gastroenterology and Nutrition, Johns Hopkins University School of Medicine, Baltimore, MD 21205, USA; E-Mail: aguerrerio@jhmi.edu; 5 Center for Celiac Research, Massachusetts General Hospital and Division of Pediatric Gastroenterology and Nutrition, Massachusetts General Hospital for Children, Boston, MA 02114, USA; E-Mail: afasano@partners.org

**Keywords:** celiac disease, gluten sensitivity, IL-10

## Abstract

Background: Intestinal exposure to gliadin leads to zonulin upregulation and consequent disassembly of intercellular tight junctions and increased intestinal permeability. We aimed to study response to gliadin exposure, in terms of barrier function and cytokine secretion, using intestinal biopsies obtained from four groups: celiac patients with active disease (ACD), celiac patients in remission (RCD), non-celiac patients with gluten sensitivity (GS) and non-celiac controls (NC). Methods: *Ex-vivo* human duodenal biopsies were mounted in microsnapwells and luminally incubated with either gliadin or media alone. Changes in transepithelial electrical resistance were monitored over 120 min. Media was subsequently collected and cytokines quantified. Results: Intestinal explants from all groups (ACD (*n* = 6), RCD (*n* = 6), GS (*n* = 6), and NC (*n* = 5)) demonstrated a greater increase in permeability when exposed to gliadin *vs.* media alone. The increase in permeability in the ACD group was greater than in the RCD and NC groups. There was a greater increase in permeability in the GS group compared to the RCD group. There was no difference in permeability between the ACD and GS groups, between the RCD and NC groups, or between the NC and GS groups. IL-10 was significantly greater in the media of the NC group compared to the RCD and GS groups. Conclusions: Increased intestinal permeability after gliadin exposure occurs in all individuals. Following gliadin exposure, both patients with gluten sensitivity and those with active celiac disease demonstrate a greater increase in intestinal permeability than celiacs in disease remission. A higher concentration of IL-10 was measured in the media exposed to control explants compared to celiac disease in remission or gluten sensitivity.

## 1. Introduction

Gluten is recognized as the environmental trigger of celiac disease (CD), an immune-mediated small intestinal enteropathy that has a prevalence in the United States of nearly 1% [1]. In this condition, the reaction to gluten’s immunogenic fraction, gliadin, is mediated by T-cell activation and is genetically associated with the human leukocyte antigen (HLA) alleles DQA1*0501/DQB1*0201. Diagnosis of CD is primarily reliant on histologic demonstration of the characteristic enteropathy, with supporting criteria, such as the presence of the celiac-specific antibodies to tissue transglutaminase (tTG) and HLA testing. Treatment of CD is lifelong maintenance of a gluten-free diet (GFD).

It is important to note that symptom alleviation on a GFD alone does not equate to a diagnosis of CD [2]. Along with the increasing awareness of CD has come the emerging recognition that there are individuals who clinically react to gluten-containing food ingestion without demonstration of a T-cell mediated process in the gastrointestinal mucosa. These individuals are classified as having non-celiac gluten sensitivity (GS) [3,4]. The rising awareness of GS is in part responsible for the growing popularity of the GFD, to the point that most people in the United States now on a GFD do not have CD [5]. While the symptoms of GS may mirror that of CD, and these symptoms may resolve on a GFD, these individuals have no histologic evidence of small intestinal enteropathy while on a gluten-containing diet. Moreover, GS carries no association with an elevation of tTG autoantibodies, although several studies have shown a higher frequency of positive anti-gliadin antibodies (AGA) than the general population [6,7,8]. While the frequency of the HLA DQ2/8 genes is higher in GS than the general population, presence of these genes, unlike in CD, is not a prerequisite [6,8]. Generally established as a “diagnosis of exclusion”, defining GS necessitates eliminating CD as the source of symptoms, followed by dietary elimination of gluten and, ultimately, reintroduction of gluten-containing foods in order to establish return of symptoms in association with gluten ingestion. This reintroduction is typically performed as an open challenge, although double-blind placebo controlled re-challenges have been performed [9,10].

The apparent lack of adaptive immune activation in GS raises the hypothesis that the similarities between GS and CD may be more related to a common defect in intestinal barrier function [11]. The intestinal barrier, by limiting uncontrolled access of non-self antigens in the lamina propria, is an integral part of immune surveillance, and impaired barrier function may play a role in a number of immune-mediated diseases, including CD [12,13,14]. In healthy intestinal epithelium, this intestinal barrier should be impermeable to macromolecules such as gliadin due to competent paracellular tight junctions. However, it has been well-demonstrated that antigenic gliadin peptides, which are inherently resistant to intraluminal digestion, are able to cross the intestinal epithelium of CD patients secondary to the gliadin-mediated, MyD88-dependent release of zonulin and consequent disassembly of the tight junction barrier [15,16]. In this study, we aimed to study whether gliadin causes a similar effect in non-celiac subjects by exposing, to gliadin, intestinal biopsy explants obtained from four different study groups: celiac patients with active disease (ACD), celiac patients in remission (RCD), non-celiac patients with gluten sensitivity (GS) and non-celiac controls (NC).

## 2. Methods

Biopsy specimens were taken from the second portion of the duodenum from adult subjects undergoing esophagogastroduodenoscopy (EGD) for clinically indicated reasons. All study subjects gave informed consent to undergo additional biopsies for the purpose of this study. This study was approved by the Institutional Review Board of the University of Maryland, Baltimore, and was conducted in accordance with their ethical standards and regulatory requirements.

### 2.1. Study Group Categorization

Clinically indicated biopsy specimens were submitted to pathology for standard reading by a pathologist with additional training in gastrointestinal pathology and were staged according to the Marsh Oberhuber classification [17]. Study subjects were classified into one of four different study groups: celiac patients with active disease (ACD, regular diet ≥ 2 months, *n* = 6), celiac patients in remission (RCD, GFD ≥ 12 months, *n* = 6), non-celiac patients with gluten sensitivity (GS, regular diet ≥ 2 months, *n* = 6), and non-celiac controls (NC, regular diet, *n* = 5).

For this study, all potential GS subjects were enrolled from the University of Maryland Center for Celiac Research in Baltimore, MD after presenting to clinic with a history of complete resolution of CD-like clinical symptoms after initiation of a GFD, yet no history of a previous EGD with duodenal biopsies while on a gluten-containing diet. To differentiate GS from CD, these patients were instructed to initiate an open (non-blinded) gluten challenge for a minimum of 2 months prior to endoscopy, with instruction to ingest a minimum of 10 grams of gluten daily, or the equivalent of 4 slices of wheat-based bread, and to subsequently proceed to endoscopy only if they experience a return of symptoms. The GS group was ultimately defined by those participants who had return of symptoms upon open gluten re-challenge and both negative CD serology (tTG and/or anti-endomysial antibodies (EMA)) and preserved duodenal villous architecture on histopathology (Marsh stage 0–1) [3]. Conversely, the ACD group was defined according to the modified 2012 criteria of the European Society of Pediatric Gastroenterology, Hepatology and Nutrition, as those participants who, after being on a gluten-containing diet for a minimum of 2 months, had biopsies demonstrating either villous blunting (Marsh 3) or, if accompanied by elevated tTG autoantibodies, crypt hyperplasia and intraepithelial lymphocytosis (Marsh 2) [18]. The RCD group was defined as having a previous biopsy-proven diagnosis of CD, with evidence of complete mucosal healing on the repeat biopsies taken at the time of the study (Marsh 0–1). These participants were maintained on a GFD prior to endoscopy. The NC group was defined as individuals that underwent an upper endoscopy because of dyspeptic symptoms; having demonstrated negative CD serology, Marsh 0–1 on duodenal histology, and no prior history of being on a GFD.

### 2.2. Transepithelial Electrical Resistance (TEER) Measurements

Intestinal permeability was assessed *ex vivo* by measuring transepithelial electrical resistance (TEER) of biopsy explants using a dual planar electrode (Endohm Evom; World Precision Instruments, Sarasota, FL), expressed in Ω cm^2^ and normalized by the baseline resistance values. After their collection, four small-intestine biopsies were oriented on presterilized filter paper with the villi facing upward and mounted onto the modified microsnapwell system as previously described by El Asmar *et al.* [19]. After 30 min incubation at 37 °C, a baseline TEER measurement (time 0) was obtained. Subsequently, a pair of biopsy explants were then luminally exposed to Dulbecco’s Modified Eagle’s Medium (DMEM) alone and a pair of biopsy explants were exposed to pepsin-trypsin digested gliadin (PT-gliadin), prepared as previously described [20] and suspended in DMEM at a final concentration of 1 mg/mL. TEER values were then monitored at 30 min intervals for a total of 120 min. TEER values for each pair of biopsies were averaged and data was expressed as the fractional change from the baseline TEER.

### 2.3. Cytokine Quantification

Media was collected from the luminal (apical) and basolateral sides of the microsnapwell system at 120 min and cytokine levels were quantified using the Human ProInflammatory 7-Plex Ultra-Sensitive Kit (Meso Scale Discovery (MSD), Rockville, MD, US) which measures interleukin (IL)-12p70, IL-1β, interferon (IFN)-γ, IL-6, IL-8, IL-10, and tumor necrosis factor (TNF)-α via multiplex electrochemiluminescence detection. MSD plates were analyzed on the MSD MS2400 imager. All standards and samples were measured in duplicate and the assays were performed according to the manufacturer’s instructions. Cytokine levels were calculated using the manufacturer’s software, given in pg/mL and presented as medians with interquartile ranges. Per the manufacturer’s instructions, the lower limit of detection, in pg/mL, for the assay is 0.77 for IL-12p70, 0.58 for IL-1β, 0.8 for IFN-γ,0.18 for IL-6, 0.10 for IL-8, 0.57 for IL-10, and 0.28 for TNF-α.

### 2.4. Statistical Analysis

Differences within each group (between explants exposed to media alone and those exposed to PT-gliadin) were assessed by Wilcoxon matched pairs signed rank test; comparisons between groups at a given time point or condition were first assessed by a Kruskal–Wallis (KW) test. For time points showing significance by KW, Mann Whitney tests were performed on all pairs. For cytokine measurements, values and differences less than one half the lower limit of detection were set to one half the lower limit of detection. All data were analyzed and graphed using Prism version 6.0 software (GraphPad Software, La Jolla, CA, USA). A *p* value < 0.05 was considered statistically significant.

## 3. Results

**Figure 1:**
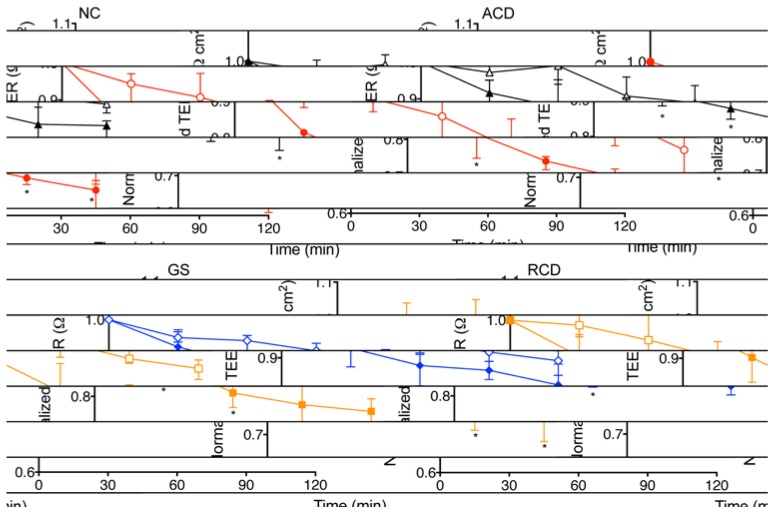
Normalized transepithelial electrical resistance (TEER) changes in human intestinal explants exposed to PT-gliadin or media alone. Explants obtained from non-celiac controls (NC), celiac patients with active disease (ACD), celiac patients in remission (RCD), and non-celiac patients with gluten sensitivity (GS). Explants were exposed to media alone (open symbols) or to PT-gliadin (filled symbols). NC (black triangle) explants demonstrated a greater increase in permeability (decrease in TEER) when exposed to PT-gliadin *vs.* exposure to media alone at 30, 60 and 120 min. RCD (blue diamond) explants demonstrated a greater increase in permeability when exposed to PT-gliadin vs exposure to media alone at 60 and 120 min. ACD (red circle) and GS (gold square) explants demonstrated a greater increase in permeability when exposed to PT-gliadin vs exposure to media alone at 30, 60, 90 and 120 min. Symbol denotes the median. Whiskers denote the 25th to 75th percentile. * *p* < 0.05.

Comparing within each group (Figure 1), there was a significant increase in permeability in intestinal explants exposed to PT-gliadin as compared to explants exposed to media alone for all time intervals (30, 60, 90 and 120 min) in the ACD group (*p* = 0.002, 0.002, 0.009, 0.04, respectively) and the GS group (*p* = 0.02, 0.009, 0.02, 0.004, respectively). In the NC group, there was a significantly greater increase in permeability in the PT-gliadin exposed explants compared to those exposed to media alone at 30, 60 and 120 min (*p* = 0.008, 0.03, 0.008, respectively). In the RCD group, there was a significantly greater increase in permeability in the PT-gliadin exposed explants compared to those exposed to media alone at 60 min only (*p* = 0.04).

**Figure 2:**
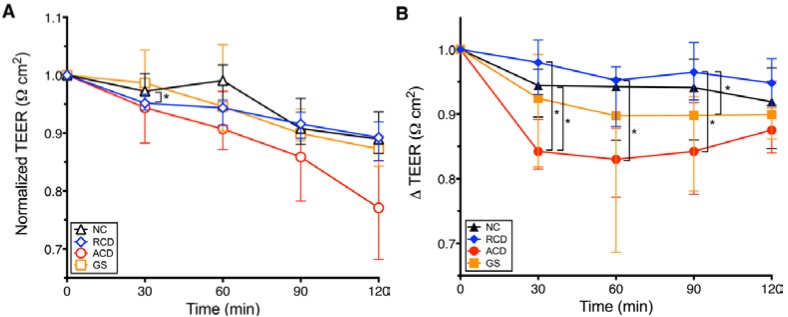
Comparison between groups of gut permeability changes induced by either PT-gliadin or media alone measured as normalized transepithelial electrical resistance changes (∆ TEER) in human intestinal explants. Explants obtained from celiac patients in remission (RCD), non-celiac controls (NC), non-celiac patients with gluten sensitivity (GS), and celiac patients with active disease (ACD). A decrease in TEER indicates increased gut permeability. A. TEER changes of explants exposed to media alone. Explants incubated in media alone demonstrated no significant differences in permeability except between NC (black triangle) and RCD (blue diamond) groups at 30 min. B. TEER of PT-gliadin exposed explants minus TEER of corresponding explants exposed to media alone (∆ TEER). Increases in permeability in explants from the ACD group (red circle) were significantly greater than in explants from the RCD group (blue diamond) at 30, 60 and 90 min, and the NC group (black triangle) at 30 min. Increases in permeability in explants from the GS group (gold square) were significantly greater than in explants from the RCD group at 90 min. There was no significant difference in permeability between explants from the ACD and GS groups, between explants from the RCD and NC groups, or between explants from the NC and GS groups. Symbol denotes the median. Whiskers denote the 25th to 75th percentile. * *p* < 0.05.

When comparing between groups, intestinal explants incubated in media alone showed no significant difference in permeability except at 30 min between the RCD and NC groups (*p* = 0.03) (Figure 2A). In order to assess for the isolated effect of exposure to PT-gliadin at each measurement interval, exposures to media alone in each study group were used as the baseline TEER values that were subtracted from the corresponding values of the PT-gliadin exposed tissue explants (Figure 2B). As shown in Figure 2B, following gliadin exposure, the increase in permeability over baseline in explants from the ACD group was significantly greater than that of the explants from the RCD group at 30, 60 and 90 min (*p* = 0.004, 0.004, 0.02, respectively). The GS group had a significant increase in permeability over baseline compared to the RCD group at 90 min (*p* = 0.03). Increase in permeability over baseline in explants from the ACD group was significantly greater than that of the explants from the NC group at 30 min (*p* = 0.02). There was no significant difference in permeability between explants from the RCD and NC groups, the NC and GS groups, or the GS and ACD groups. There was no significant difference in permeability between explants from the RCD and NC groups, the NC and GS groups, or the GS and ACD groups. Coefficient of variation (mean ± standard deviation) for the TEER measurements were 16% ± 20%, 8.5% ± 6.5%, 2.8% ± 1.3%, and 6.4% ± 5.6% for the GS, ACD, RCD, and NC samples, respectively, in media alone, while it was 11% ± 10%, 17% ± 14%, 5.8% ± 3.6%, and 5.1% ± 3.2%, respectively, in the media plus PT-gliadin samples.

**Figure 3:**
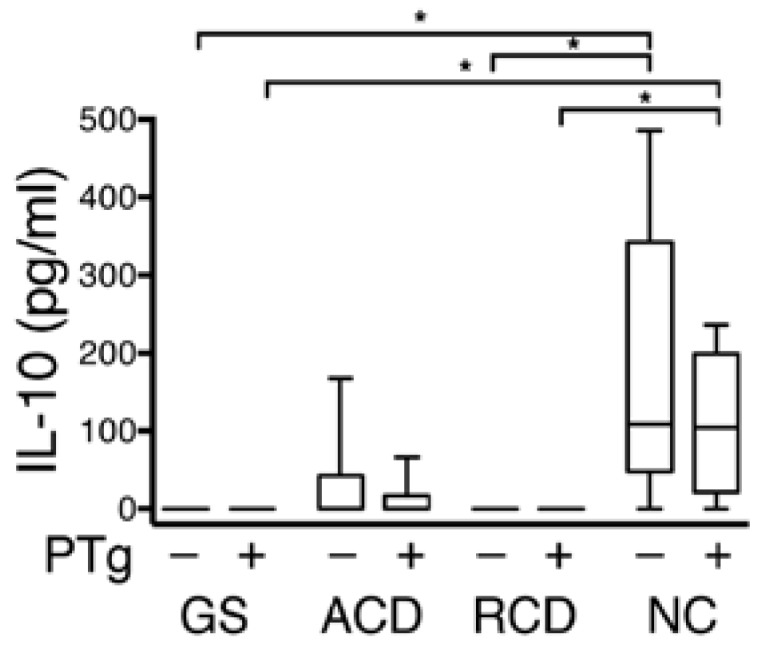
Comparison between groups of IL-10 secretion from the basolateral side of biopsy explants after 120 min, with and without PT-gliadin exposure. Media collected from the basolateral sides of explants obtained from non-celiac patients with gluten sensitivity (GS), celiac patients with active disease (ACD), celiac patients in remission (RCD), and non-celiac controls (NC). The NC group showed concentrations of IL-10 significantly higher than the corresponding media from the GS and RCD group. In the graphs, the box defines the 25th and 75th percentile; center line the median; whiskers the range. * *p* < 0.05.

After 2 h, production of the anti-inflammatory cytokine IL-10 was significantly greater for the NC group compared to the GS and RCD groups in media alone (*p* = 0.01, 0.03) and in PT-gliadin containing media collected from the basolateral side of explants (*p* = 0.01, 0.03) (Figure 3). There was no significant difference in the change of measured levels of the proinflammatory cytokines IL-12p70, IL-1β, IFN-γ, IL-6, IL-8, or TNF-α on exposure to PT-gliadin in either the luminally or basolaterally exposed media and in IL-10 in the luminally exposed media (data not shown).

## 4. Discussion

CD is mediated by T-cell activation against gluten’s immunogenic fraction, gliadin; specifically, a 33-mer peptide fragment that is resistant to intraluminal proteolysis and serves as the primary antigen for T-cell proliferation [21]. Activation of the adaptive immune response requires specific HLA class II genes and is typically accompanied by specific celiac-associated IgA antibodies; the end result of this inflammatory cascade—the characteristic enteropathy—must be histologically demonstrated for a diagnosis of CD to be made. However, there is an emerging recognition that there are individuals who react to gluten-containing food in a manner symptomatically indistinguishable from CD yet without the typical CD serology or associated histopathology. These individuals are defined as having non-celiac gluten sensitivity (GS) [3,4]. Adverse reactions to gluten outside the gastrointestinal tract, such as dermatitis herpetiformis and gluten ataxia [22], have been well-studied, yet research has only recently begun to clearly define the spectrum of gastrointestinal gluten-related disorders, and there are no objective laboratory markers to-date that are specific for GS. As the absence of an adaptive immune response in GS distinguishes this condition from CD, the aim of this study was to further evaluate the role of the intestinal barrier in these two conditions, both in terms of its barrier function and cytokine secretion.

Due to the integrity of intestinal tight junctions, normal intestinal epithelium should be impermeable to such indigestible macromolecules such as the 33-mer gliadin peptide fragment, yet in CD these antigens are able to pass from the lumen to the lamina propria. Compromised permeability to macromolecules appears to be independent of the enteropathy encountered in CD, with research demonstrating that unaffected first-degree relatives of CD patients similarly have increased intestinal permeability at baseline [23]. The mechanisms of gliadin-induced permeability in CD have been well-studied and include the interaction of specific gliadin peptides with the CXCR3 receptor expressed on the luminal side of enterocytes, paracellular trafficking via zonulin release, and subsequent tight junction disassembly [15,16,24,25]. Enhanced intestinal permeability upon gliadin exposure does not appear to be limited to CD patients. A previous *ex vivo* study conducted by Drago *et al.* using duodenal biopsy explants in a microsnapwell system demonstrated that, following 60 min of PT-gliadin exposure, tissue from both RCD patients and controls demonstrated a significant increase in intestinal permeability as well as a temporally-associated increase in zonulin release [20]. Moreover, a recently published study by Vazquez-Roque *et al.* using lactulose/mannitol (LA/MA) permeability testing in patients with diarrhea-predominant irritable bowel syndrome (IBS) demonstrated higher small bowel permeability in patients randomized to a 4-week gluten-containing diet compared to those randomized to a four-week GFD [26].

In order to investigate the role of gut barrier dysfunction in gluten-related disorders, a study conducted by Sapone *et al.* performed LA/MA permeability testing on GS, ACD and dyspeptic (control) patients [8]. The ACD group demonstrated a significantly higher urinary LA/MA ratio (increased intestinal permeability) than the GS group. However there was not an associated increase in the urinary LA/MA ratio when compared to the controls in either the ACD or the GS population. This urinary LA/MA ratio was performed on patients after a period of fasting and, as such, is a marker of baseline permeability. In our *ex vivo* study, this is akin to the baseline, or media-only, TEER study arm shown in Figure 2A. Like the Sapone *et al.* study, our TEER results of the media-only biopsy explants demonstrated no increase in permeability in the GS group compared to the NC group, or in the ACD group compared to the NC group.

Figure 1 and Figure 2B illustrate the effects on intestinal permeability after direct exposure to PT-gliadin. Similar to the previously mentioned study on RCD and control patients conducted by Drago *et al.* we found a statistically significant increase in intestinal permeability upon PT-gliadin exposure across all four study groups (Figure 1). Figure 2B demonstrates that, when comparing between groups, upon PT-gliadin exposure, the GS explants reacted similarly to the ACD explants, with no statistical difference between these groups. The NC explants reacted similarly to the RCD explants, likewise with no statistical difference between these groups. Both the ACD and GS explants had a statistically greater increase in permeability than the RCD explants, and explants from the NC and RCD groups reacted to a lesser degree than those from the ACD group. These results complement those previously reported by Sapone *et al.*; although GS patients have a normal intestinal barrier at baseline, gliadin exposure induces increased intestinal permeability in these patients, with a response that closely resembles that observed in the ACD group and is more pronounced than that observed in the RCD group.

The RCD group demonstrated the smallest degree in permeability change upon exposure to PT-gliadin and the difference reached significance at only 60 min. This is a delay when compared to the other study groups, which all demonstrated an increase in permeability at the first measured interval of 30 min. The RCD group was the only group in our study on a strict GFD prior to endoscopy, suggesting that the gluten-induced activation of the zonulin pathway is comparatively delayed in intestinal tissue that is not routinely exposed to dietary gluten, even in those patients with celiac disease. Similarly, the randomized controlled trial by Vazquez-roque *et al.* demonstrated significantly increased small bowel permeability by (LA/MA) testing in IBS patients randomized to a gluten-containing diet compared to those randomized to 4 weeks of a strict GFD [26]. Given that symptom-onset upon gluten-containing food exposure in the GS population is comparatively faster than in patients with CD, further permeability experiments comparing GS patients on a strict GFD to those with RCD would help contribute to the understanding of the timing of these alterations.

Cytokine quantification from the media collected from the luminal and basolateral sides of the biopsy explants after 120 min of PT-gliadin exposure failed to show any significant difference in the classic inflammatory cytokines, to include IL-6, IL-8, IFN-γ, and TNF-α. The relatively short period of incubation may in part explain the lack of detection of an inflammatory innate response to gluten exposure. Production of the anti-inflammatory cytokine IL-10 was significantly higher in the media collected from the basolateral side of the explants obtained from the NC group as compared to the GS and RCD groups (Figure 3) in both the unexposed media and the media exposed to PT-gliadin. IL-10 is a key mediator for regulating the innate intestinal immune response [27]. A study by Madsen *et al.* in IL-10 deficient mice demonstrated that IL-10 plays a significant role in intestinal permeability and, in fact, deficiencies of IL-10 lead to increased intestinal permeability prior to the development of mucosal inflammation [28]. We do not suggest that patients with GS or CD are IL-10 deficient; however, this degree of IL-10 production in the NC group may be indicative of a more competent innate immune response. Further studies will be needed to help elucidate if this lack of secretion of IL-10 is a primary factor leading to an exaggerated increase in PT-gliadin induced permeability or a secondary marker of a separate defect in innate immunity.

A primary limitation of this study relates to the lack of objective laboratory biomarkers specific for GS. Currently, the diagnosis is based on exclusion criteria; specifically these patients should have negative CD serology (anti-EMA and/or anti-tTG), normal duodenal histopathology, and resolution of CD-like symptoms on a GFD with subsequent return of symptoms upon dietary reintroduction of gluten-containing food [3]. For the purposes of this study, our GS patients had undergone an open gluten challenge and we recognize that such a method could allow for the possibility of patients without true GS being included in the GS group due to placebo-response. While dietary re-challenge in a blinded fashion would have helped avoid the possibility of a placebo effect, the necessity of a gluten-containing diet at biopsy precluded this type of intervention. Similarly, no patients in our control group had previously attempted a GFD; ideally, the control group would have been comprised of those who had already had GS ruled-out by a negative GFD trial. The percentage of patients with IBS who truly have GS may be as high as 30%; given that the indication for duodenal biopsies in our NC group would include the non-specific gastrointestinal symptoms of CD and GS, it is conceivable that some of the NC patients may have undiagnosed GS [9].

## 5. Conclusions

This study demonstrates that gliadin exposure induces an increase in intestinal permeability in all individuals, regardless of whether or not they have celiac disease. The results of this study suggest that gluten exposure leads to altered barrier function in both ACD and GS, resulting in an exaggerated increase in intestinal permeability when compared to RCD. The intestinal mucosal secretion of IL-10 from the basolateral surface seen in NC subjects in this study was not observed in those with RCD or GS. Specific laboratory markers for GS are still necessary to allow for a more objective definition of GS and further research into GS disorders would benefit from double-blind, placebo-controlled studies.
